# Liposomal Delivery of Saquinavir to Macrophages Overcomes Cathepsin Blockade by *Mycobacterium tuberculosis* and Helps Control the Phagosomal Replicative Niches

**DOI:** 10.3390/ijms24021142

**Published:** 2023-01-06

**Authors:** David Pires, Manoj Mandal, Jacinta Pinho, Maria João Catalão, António José Almeida, José Miguel Azevedo-Pereira, Maria Manuela Gaspar, Elsa Anes

**Affiliations:** 1 Host-Pathogen Interactions Unit, Research Institute for Medicines, iMed-ULisboa, Faculty of Pharmacy, Universidade de Lisboa, Av. Prof. Gama Pinto, 1649-003 Lisboa, Portugal; 2 Center for Interdisciplinary Research in Health, Católica Medical School, Universidade Católica Portuguesa, Estrada Octávio Pato, 2635-631 Rio de Mouro, Portugal; 3 Advanced Technologies for Drug Delivery, Research Institute for Medicines, iMed-ULisboa, Faculty of Pharmacy, Universidade de Lisboa, Av. Prof. Gama Pinto, 1649-003 Lisboa, Portugal

**Keywords:** tuberculosis, phagosomal niches, survival strategies, cathepsins, saquinavir, protease inhibitors, liposomes, host directed therapies

## Abstract

*Mycobacterium tuberculosis* is able to establish a chronic colonization of lung macrophages in a controlled replication manner, giving rise to a so-called latent infection. Conversely, when intracellular bacteria undergo actively uncontrolled replication rates, they provide the switch for the active infection called tuberculosis to occur. Our group found that the pathogen is able to manipulate the activity of endolysosomal enzymes, cathepsins, directly at the level of gene expression or indirectly by regulating their natural inhibitors, cystatins. To provide evidence for the crucial role of cathepsin manipulation for the success of tuberculosis bacilli in their intracellular survival, we used liposomal delivery of saquinavir. This protease inhibitor was previously found to be able to increase cathepsin proteolytic activity, overcoming the pathogen induced blockade. In this study, we demonstrate that incorporation in liposomes was able to increase the efficiency of saquinavir internalization in macrophages, reducing cytotoxicity at higher concentrations. Consequently, our results show a significant impact on the intracellular killing not only to reference and clinical strains susceptible to current antibiotic therapy but also to multidrug- and extensively drug-resistant (XDR) Mtb strains. Altogether, this indicates the manipulation of cathepsins as a fine-tuning strategy used by the pathogen to survive and replicate in host cells.

## 1. Introduction

Tuberculosis (TB) has afflicted humankind since the ‘Out-of-Africa’ migrations of *Homo sapiens* [1]. Consequently, the main causative agent, *Mycobacterium tuberculosis* (Mtb), is among the most well-adapted human pathogen species [2]. In fact, it is estimated that about one quarter of the human population is latently infected (LTB) [3]. LTB is defined as a state of persistent immune response to Mtb antigens without evidence of clinically manifested active disease [4]. In the aforementioned population, approximately 600,000 people are estimated to be carriers of multidrug-resistant (MDR) and extensively drug-resistant Mtb strains (XDR) [3]. About five to ten percent of this latent infected population will develop TB following immunosuppressive conditions, e.g., of HIV coinfection or comorbidities such as diabetes, aging, or malnutrition. TB has a high prevalence in developing countries, where poor health conditions and poor access to food and medication accelerate reactivation from dormancy and the spread of the disease. With about two million deaths annually and the increased multidrug resistance, the World Health Organization (WHO) has declared TB a global emergency since 1992 [5].

Mtb spreads by inhalation of bacteria containing aerosols and infects lung macrophages through the airways [6]. The bacterium internalized in these professional phagocytic cells can, to some extent, circumvent their potent bactericidal mechanisms and survive and replicate intracellularly, establishing a successful chronic infection [7,8,9,10,11,12,13]. Therefore, deciphering the pathways that grant Mtb the ability to overcome the effective killing mechanisms, which allow the establishment of permanent intracellular niches in macrophages, is required to control TB.

In recent years, our group has been devoted to investigating the involvement of lysosomal cathepsins during host Mtb interactions and their relevance during innate and adaptive immune responses [14,15,16,17,18]. Cathepsins are major players integrated into most processes associated with the lysosome [19,20,21,22]. In this organelle, they are involved in direct pathogen digestion and killing [14], protein degradation [23], autophagy [24], the processing of antigens to peptides and their presentation via human leukocyte antigen class II (HLA-II) machinery [25], cellular stress signaling, and lysosome-mediated cell death [26]. In addition to the endocytic pathway, they are also relevant to control infections in other cellular spatial localizations or in the extracellular environment as recently reviewed [27].

We previously found that during the infection of human macrophages, Mtb manipulates cathepsins, inducing a general downregulation of gene expression in parallel with a decreased protease activity, either in resting M0 or M1 proinflammatory polarized macrophages [14]. Relevant targets to manipulate the proteolytic activity of cathepsins include microRNAs [28], namely miR-106b-5p targeting cathepsin S [15], and cathepsin natural inhibitors, such as cystatin C [16]. Recently, we found that another protease inhibitor used in antiretroviral therapy for HIV infection, saquinavir [29], not only inhibits the viral protease, compromising the maturation and infectivity of new viral particles, but is also able to activate the bulk of cathepsin proteolytic activity during Mtb infection of macrophages [18]. In this study, we demonstrate that saquinavir (SQV) incorporated in liposomes increases the efficiency of the HIV protease inhibitor internalization in macrophages, reducing their cytotoxicity at higher concentrations while impacting the proteolytic activity of cathepsins. By using this strategy, we aim to demonstrate that cathepsins are major players during Mtb infection and that overcoming the cathepsin activity blockade induced by the pathogen may contribute to controlling the infection.

## 2. Results

### 2.1. A Negatively Charged Lipid Composition Results in High Load of SQV Incorporation in Liposomes

As was mentioned before, HIV protease inhibitors, specifically SQV, were previously shown to impact human lysosomal proteases [18,30]. To increase the effectiveness of SQV and to provide evidence of the crucial role of cathepsin manipulation for the success of tuberculosis bacilli in their intracellular survival, we decided to encapsulate SQV in a liposomal delivery system. The rationale for this approach is based on the fact that phagocytic cells efficiently internalize particles with sizes >0.1 μm, as is the case of liposomes, thus allowing loaded compounds delivery to infected cells at a high extent. In fact, the incorporation of antibiotics in negatively charged nanoformulations [31] has been effectively used both in tuberculous [32] and non-tuberculous infections [33]. Consequently, we decided to evaluate the loading of SQV in different negatively charged or neutral lipid compositions. We tested three formulations: dimiristoyl phosphatidyl choline (DMPC) and dimiristoyl phosphatidyl glycerol (molar ratio 8:2); the lipid composition selected in the present work, composed of dioleoyl phosphatidyl choline (DOPC) (both negatively charged liposomal formulations) and dioleoyl phosphatidyl glycerol (DOPG); and dioleoyl phosphatidyl choline (DOPC) and dioleoyl phosphatidyl ethanolamine (DOPE) (molar ratio 8:2) (a neutral charged liposomal formulation). In all tested lipid compositions, the main choline phospholipid was always used at 80 mol%, namely DMPC, DOPC.

The preliminary results allowed us to conclude that a negatively charged liposomal formulation was better, particularly the one constituted by phospholipids with lower phase transition temperature (Tc), as in the case of DOPC (Tc = −17 °C), when compared to DMPC (Tc = +23 °C).

Table 1 shows the formulation composed of DOPC: DOPG that we selected because it displayed high SQV incorporation and because preliminary results indicated a high SQV internalization in macrophages. Indeed, SQV liposomes with a mean size ≈ 100 nm are highly homogenous, as demonstrated by the low polydispersion index (P.I.), presented E.E. superior to 90%.

### 2.2. SQV-Loaded Liposomes Are Effectively Internalized by Mtb-Infected Macrophages

To test the efficacy of liposomal delivery of SQV to host macrophages, we analyzed the capacity of Mtb-infected cells to incorporate rhodamine-labelled SQV liposomes (Table 1). Macrophages infected with Mtb H37Rv were treated for 3 h with selected SQV liposomes (LipSQV) and then analyzed by flow cytometry and fluorescence microscopy. The SQV concentrations tested (ranging from 10 to 50 µg/mL) were selected based on prior studies by us and others [18,34,35] on the pharmacokinetics of the drug [30] and on the objective to increase the upper limit concentration tolerated without cytotoxic effects.

**Figure 1:**
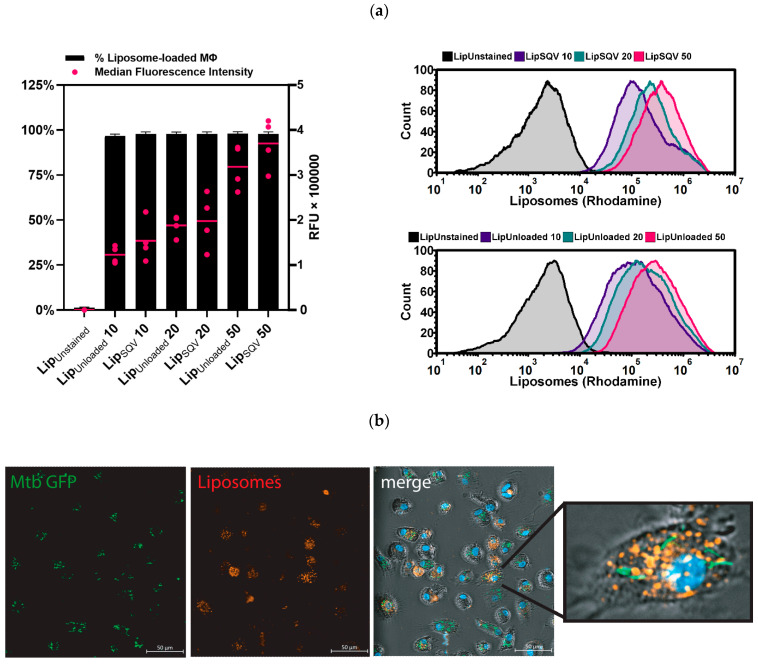
SQV-loaded liposomes are effectively internalized by Mtb-infected human macrophages. (**a**) Flow cytometry analysis of rhodamine-labeled liposomes incorporated by macrophages infected with Mtb H37Rv after 3 h of treatment. The bars represent the mean percentage of macrophages positive for liposomes (left *y*-axis). The dots represent the median fluorescence intensity per macrophage for each donor (right *y*-axis) and the horizontal lines represent the mean of all donors. The histogram displays one representative experiment. Error bars depict the standard error of the mean calculated from 4 independent experiments. RFU—relative fluorescence units. (**b**) Fluorescence microscopy analysis of the same conditions analyzed in (**a**). Mtb H37Rv is depicted in green and SQV-loaded liposomes are depicted in red; the nucleus is contrasted in blue.

The results show that for all concentrations assayed, almost 100% of the population of macrophages incorporated LipSQV (Figure 1). Variations between treatments were mostly observable by flow cytometry analysis of the median fluorescence intensity (Figure 1a), indicating that on average LipSQV50 treatment resulted in macrophages with twice the fluorescence intensity of LipSQV20, with the latter having 25% more intensity than LipSQV10. These results indicate that while LipSQV uptake is widespread even at lower concentrations, there is a relevant increase in the number of liposomes uptaken when higher concentrations are applied. Furthermore, fluorescence microscopy analysis (Figure 1b) shows higher liposome uptake by infected macrophages.

### 2.3. SQV-Loaded Liposomes Present No Cytotoxicity at Therapeutic Concentrations

**Figure 2:**
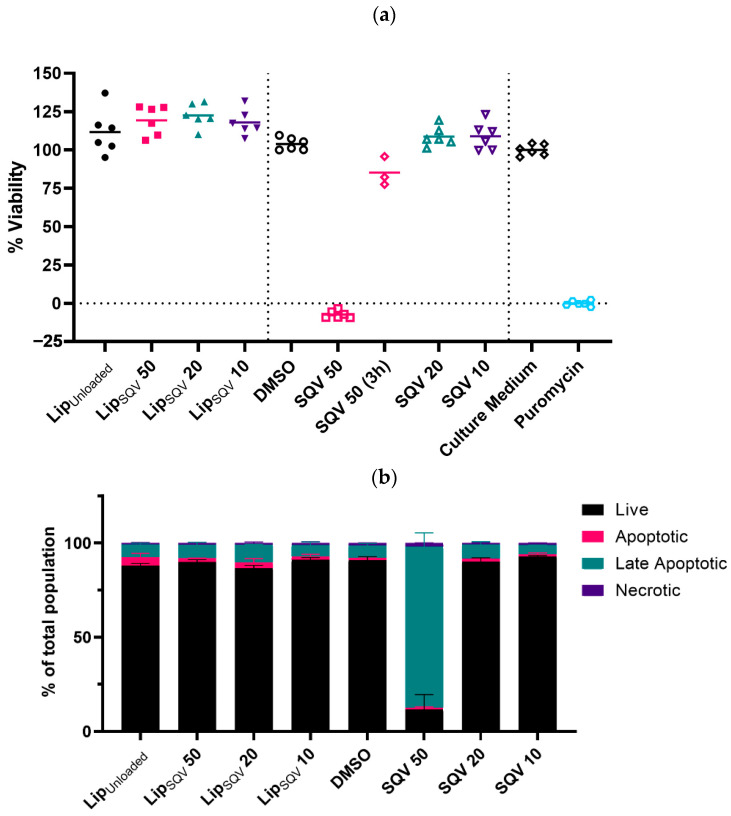
SQV-loaded liposomes present no cytotoxicity for human macrophages after 3 days of treatment. (**a**) Macrophages were treated with the selected concentrations of LipSQV or free SQV and incubated with the drugs for 3 days. Additionally, free SQV was tested at 50 µg/mL for only 3 h. Macrophage viability was measured using PrestoBlue (resazurin-based solution) by quantifying the fluorescence emission in a plate reader. LipUnloaded or DMSO were used as the negative controls and puromycin (5 µg/mL) as the positive control for cell death. The symbols depict the relative viability of cells from different donors compared to cells treated with culture medium alone. (**b**) Cell death was measured by flow cytometry analysis of Mtb-infected macrophages following 3 days of treatment, using Apotracker Green and Zombie Red viability dyes. Bars represent the average of two independent experiments and the error bars depict the standard error of the mean.

SQV therapeutic application is highly influenced by its cytotoxicity in moderate concentrations [18,36], difficulty in maintaining therapeutic levels in plasma [37], and the known negative interactions with the current anti-TB drug rifampicin [38]. Having established that the LipSQV formulations are effectively uptaken by infected macrophages, we assessed the cytotoxic effects of SQV using this nanoformulation. For this, we first analyzed non-infected macrophages treated with the selected concentrations of LipSQV and compared the results with cells treated with free SQV for 3 days (Figure 2a). This longer period of incubation served to confidently determine the effects of SQV exposure otherwise under-evaluated using a 3 h short treatment applied systematically to cytotoxicity assays. By using resazurin assays, the results show no cytotoxicity in all conditions tested, apart from an observed strong decreased viability of the cells during treatment with free SQV at 50 µg/mL. Remarkably, the treatment of cells using liposomal-loaded SQV at the same concentration of the drug resulted in no effect on macrophage viability. 

These results were confirmed by flow cytometry in Mtb-infected cells by quantifying the impact of the treatments on programmed cell death following 3 days of incubation (Figure 2b). Apotracker Green and Zombie Red were used as markers for apoptosis and necrosis, respectively. These dyes allow the fixation of the samples following staining and thus permit flow cytometry cell death analysis outside the BSL-3 laboratory. The results show no impact on cell death during all tested conditions, with the exception of free SQV at 50 µg/mL (Figure 2a,b). 

To further establish the conditions for the following experiments, we decided to test whether a 3 h treatment using free SQV at 50 µg/mL could induce similar cytotoxicity. Following this treatment, cells were washed and incubated until day 3 without additional contact with the free drug. As shown in Figure 2a, the results indicate an average viability of 85%. We thus decided to establish a 3 h incubation for the following experiments when comparing the treatment of LipSQV with the free drug.

### 2.4. SQV-Loaded Liposomes Significantly Improve the Free-SQV Ability to Increase Cathepsin Activity

**Figure 3:**
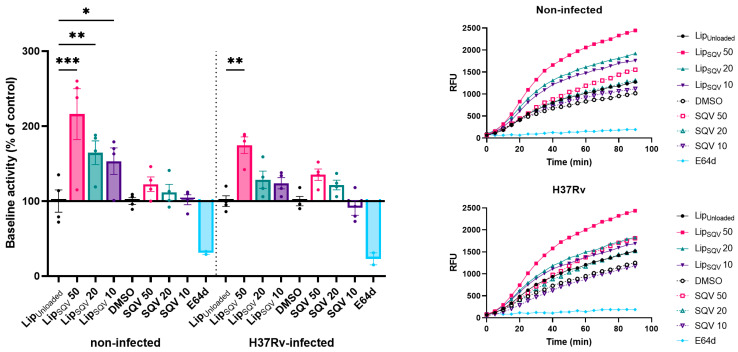
SQV-loaded liposomes increase the proteolytic activity of cathepsins in Mtb-infected human macrophages. Mtb-infected macrophages were treated for 3 h, then washed and incubated without drugs for an additional 24 h. Following that period, the general activity of cathepsins was monitored using a fluorogenic substrate and measured every 5 min for 90 min. General inhibitor of cathepsins, E-64d, was used as a negative control. Bars represent average baseline activity of four independent experiments. The symbols represent each donor tested and the error bars depict the standard error of the mean. Baseline activity was calculated as the maximum slope of fluorescence emission over 1 h. LipUnloaded and DMSO were used as references for LipSQV and free SQV treatments, respectively. Line plots display average fluorescence per time. * *p* ≤ 0.05, ** *p* ≤ 0.01, *** *p* ≤ 0.001. RFU—relative fluorescence units.

Following the determination of the experimental conditions that produced a minimal effect on cell viability, we assessed the impact of the treatments in the proteolytic activity of cathepsins. General cathepsin activity was measured for 1.5 h using a fluorogenic peptide substrate specific for several cathepsins, including cathepsins B, L, and S. The inhibitor of most cathepsins, E-64d, was used as a negative control [39]. For these experiments, we performed a 3 h treatment with the drugs, then washed the cells and incubated them for a further 24 h without additional treatments. Since we and others have already demonstrated SQV impact on human cathepsin activity [18,28], we now sought to verify whether similar activity could be maintained 24 h after SQV or LipSQV removal from the culture medium. Indeed, the results show a marked increase in cathepsin proteolytic activity (Figure 3) in non-infected macrophages treated with SQV in liposomal form for 3 h, at 50, 20, and 10 µg/mL, in contrast to all conditions assayed with the free drug.

When using Mtb-infected macrophages, only for the highest concentration (the LipSQV at 50 µg/mL), a statistically significant impact on cathepsin proteolytic activity was observed, confirming the strong inhibition of cathepsin activity induced by the pathogen. These results solidify the additional benefit of liposomal encapsulation of SQV in prolonging the effects on proteolytic activity in non-cytotoxic conditions and the impact during Mtb infection.

### 2.5. SQV-Loaded Liposomes Improve the Intracellular Killing of Mtb Reference Laboratory and Clinical Strains with Different Drug Resistance Profiles

**Figure 4:**
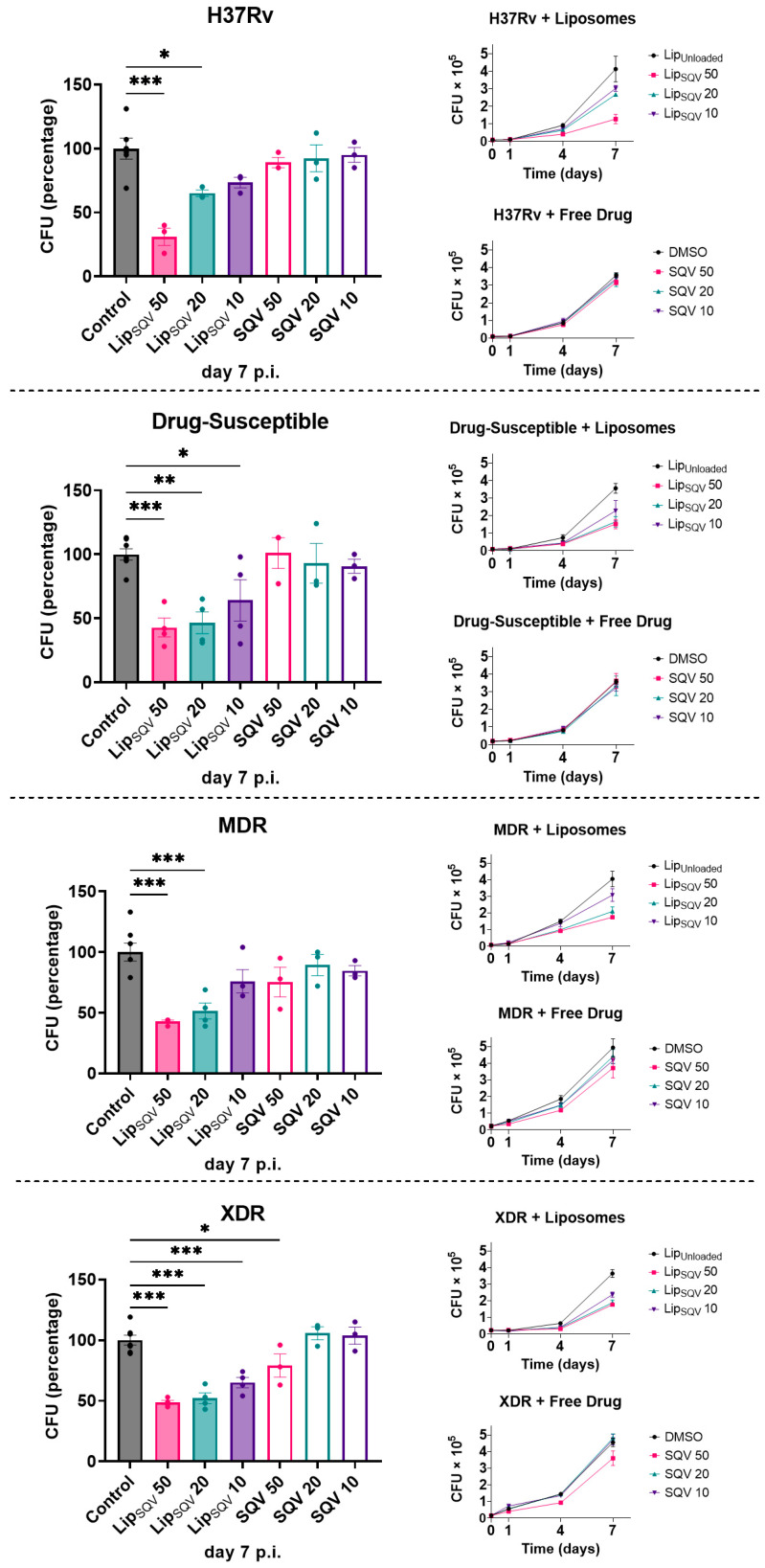
SQV-loaded liposomes improve the intracellular killing of Mtb laboratorial and clinical strains with different drug resistance phenotypes in human macrophages. Macrophages were infected with different Mtb strains for 3 h and then treated with selected concentrations of LipSQV and free SQV. LipUnloaded and DMSO were used as controls. To evaluate bacterial intracellular survival, at discrete time-points, macrophages were lysed, and serial dilutions of the bacterial suspension were plated on 7H10 agar plates. Colony-forming units were counted following 2–3 weeks. Lines depict average CFU per sample from at least 3 independent experiments. Bars represent average CFU relative to the respective controls at day 7 post-infection. Symbols represent each experiment with macrophages from a different donor. Error bars represent the standard error of the mean. * *p* ≤ 0.05, ** *p* ≤ 0.01, *** *p* ≤ 0.001.

Saquinavir was already demonstrated to improve the intracellular killing activity of Mtb by human macrophages as a consequence of the increased proteolytic activity [18]. In those studies, and in order to achieve the greatest effects, macrophages were incubated with SQV prior to infection using 20 µg/mL, the highest concentration that was shown to provide no cytotoxic effects. Here, our hypothesis is that, by using liposomes as a vehicle, those results may be achieved without the requirement of a pre-incubation. Indeed, we do expect that by reducing the cytotoxic effects of SQV we can use higher concentrations previously unattainable. We therefore assessed the Mtb intracellular killing by macrophages using the conditions established in the previous sections and measured the colony-forming units (CFU) of bacteria recovered from infected cells over a period of 7 days (Figure 4). To account for strain-specific effects, and to test the effectiveness of the treatments in strains that accumulated mutations conferring drug resistance, we compared the effects of treatments using the H37Rv reference strain with clinical strains isolated from patients with tuberculosis. All were typified by the national reference institute for health (INSA) concerning antibiotic susceptibility. Here, we selected strains that were drug-susceptible, multidrug resistant (MDR), and extensively resistant (XDR) to antibiotics. The results indicate that LipSQV was able to significantly impact the macrophages’ killing ability to all strains in a dose-dependent manner, as shown in Figure 4. However, no effects on Mtb killing were observed when using the free drug, except for the XDR strain for the SQV highest concentration of 50 µg/mL.

## 3. Discussion

One key aspect of Mtb success in humans is its ability to establish a replicative niche in phagocytes, particularly macrophages [7,10,11], the same cells that contribute to their control and elimination. Seminal studies by D’Arcy Hart [8] in 1975 had already mentioned that Mtb promote their survival within the host by acting from within phagosomes to prevent phagolysosome biogenesis and shield the bacteria from being exposed to lysosomal hydrolases. Among these hydrolases, cathepsins are the most represented proteases of the endolysosomal pathway, with a crucial role in pathogen killing and in the elimination of cell debris [40,41]. It is not unlikely that intracellular pathogens such as Mtb evolved strategies to manipulate these enzymes to avoid the activation of the microbicidal mechanisms.

Indeed, we recently explored the different strategies that Mtb may use to prevent their digestion by cathepsins. We found different levels of manipulation of these enzymes induced by Mtb, leading to a general down-regulation of cathepsin gene expression, protein translation, and enzymatic activity [14,15]. This was particularly noticeable for cathepsins B, L, and S, which are among the most expressed in macrophages. Remarkably, this phenotype could not be replicated using non-pathogenic mycobacteria, suggesting its specific relevance to Mtb intracellular success. Regarding these observations, we have recently explored SQV’s potential as a repurposed drug that enhances macrophages’ intracellular response to Mtb infection [18]. SQV was one of the first drugs developed to control HIV infection as part of combined antiretroviral therapy [42]. It blocks the cleavage of the Gag-Pol protein precursor by inhibiting HIV protease. This prevents the full maturation of nascent virions and their ability to infect new cells [43,44,45]. Acting as an aspartic protease inhibitor, it is likely that SQV could interfere with other pathogens that depend on proteases for their life cycle or that depend on protease manipulation of the target cells they infect. Some studies have already reported the inhibitory activity of SQV against a diverse array of pathogens, such as the virus SARS-CoV and avian influenza [46], the fungi *Fonsecae pedrosoi* [47], and the parasites *Trypanosoma cruzei* [48] and *Plasmodium falciparum* [49]. 

Saquinavir’s interaction with other proteases does not appear to be restricted to microorganisms. Aspartyl proteases such as cathepsin D and E, or cysteine proteases such as cathepsin S, were also altered by the protease inhibitors [30]. In the case of Mtb infection, we found that SQV restores and further improves the overall activity of cathepsins in Mtb-infected macrophages and more specifically, that of cathepsin S. One caveat of that study was the requirement to pre-treat macrophages with SQV to achieve the greatest effects and maintain the treatment throughout the infection. This would not be feasible to translate to the clinical setting. Moreover, constant exposure of macrophages to moderate doses of SQV as low as 20 µg/mL often induces cytotoxic effects.

In the present work, these limitations were overcome using SQV loaded in liposomes. Our results indicate a high internalization of the HIV protease inhibitor in macrophages while reducing its cytotoxicity at higher concentrations and effectively impacting the proteolytic activity of cathepsins relative to free drug treatment. Moreover, we could achieve similar benefits of continuous treatment, restricting the exposure to the drug to only 3 h. This proved to be beneficial for the host macrophage microbicidal effects, enhancing Mtb intracellular proteolytic digestion. 

Another potential limitation of the usage of SQV for tuberculosis treatment is the known interaction with rifampicin, resulting in a marked reduction in patients’ serum levels [50,51]. Liposome-based delivery of SQV might constitute a solution to overcome these challenges by shielding SQV from rifampicin-induced drug metabolism. In fact, we observed the absence of cytotoxic effects when using liposomes to deliver concentrations of SQV much higher than those safely achieved in the serum of patients treated with the free drug. In comparison, the same concentrations of free SQV resulted in 85% apoptosis after 3 days of treatment. Notably in this context, tuberculosis is responsible for almost 30% of all deaths of HIV-infected patients [3], and any improvement to the current ability to treat both infections with minimal adverse effects could have a significant impact on these individuals.

The results indeed demonstrated the efficacy of SQV-loaded liposomes in contributing to control infections by Mtb clinical strains susceptible or resistant to the current antibiotic therapy. However, a more extensive liposomal characterization is needed to clarify the real drug concentration released into macrophages, allowing a possible application of liposomes in a clinical setting.

The incidence of multidrug-resistant and rifampicin-resistant tuberculosis reached 450,000 new cases in 2021, nearly 4% of all TB cases in that year [3]. Resistance to isoniazid and rifampicin severely complicates treatment, increasing its length by up to 20 months and reducing its success rate to 60% [3]. Clinical application of recently developed antibiotics such as bedaquiline is being pushed by the WHO to improve the treatment of these drug-resistant cases and significantly lower the duration of treatment [52,53]. This demonstrates how the introduction of new drugs could favorably impact the treatment of the most difficult cases. Our results with liposomal delivery of SQV show similar levels of activity between all strains tested, irrespective of their drug resistance profile. 

Altogether, these results demonstrate that although Mtb appears to manipulate cathepsins as a fine-tuned survival strategy, we provide evidence for how to overcome the pathogen-induced blockade to impact the capacity of macrophages to control the infection.

In addition, we propose a host-directed therapy using the repurposed antiviral drug saquinavir to target the intracellular replicative niche of Mtb. This may be applied as a complementary strategy to current antibiotics to increase the efficacy of treatment, lower its toxicity and drug interactions, while simultaneously delivering lower selective pressure for the evolution of drug-resistant strains.

## 4. Materials and Methods

### 4.1. Preparation and Physicochemical Characterization of Saquinavir Liposomes

The encapsulation of saquinavir (SQV) in liposomes was performed by an active loading method with an ammonium sulphate gradient as previously described [54,55]. Briefly, the relevant phospholipids, dioleoyl phosphatidyl choline (DOPC), and dioleoyl phosphatidyl glycerol (DOPG), purchased from Avanti Polar Lipids, at a molar ratio of 8:2 and a lipid concentration of 30 µmol/mL, were dissolved in chloroform, and the organic solvent was removed by rotary evaporation. The formed homogeneous lipid film was hydrated with water and the so-formed suspension was frozen (−70 °C) and lyophilized in a freeze-dryer (Edwards, CO, USA) overnight. The rehydration of the lyophilized powder was performed with ammonium sulphate and trehalose (60 mM and 5%, respectively), pH 5.4 at room temperature for 30 min. To produce a homogeneous liposomal suspension, unloaded liposomes were filtered under nitrogen pressure (10–500 lb/in2) through polycarbonate membranes of proper pore size using a Lipex thermo-barrel extruder (Lipex: Biomembranes Inc., Vancouver, BC, Canada) until liposomes with a mean size of around 0.1 µm were attained. An ammonium sulphate gradient was created by replacing the extraliposomal medium with phosphate-buffered saline (PBS) buffer (pH 8.4), the pKa for SQV, using a Econo-pac 10 DG desalting column (Bio-Rad Laboratories, Hercules, CA, USA). SQV was incubated with unloaded liposomes at a final concentration of 50 µg / µmol of lipid for 60 min at room temperature. At this stage, a sample of the suspension was taken for further quantification in terms of SQV and lipid contents corresponding to initial conditions. The non-encapsulated SQV was separated by ultracentrifugation at 250,000× *g* for 2 h at 15 °C in a Beckman LM-80 ultracentrifuge (Beckman Instruments, Inc., Fullerton, CA, USA). The pellet was suspended in PBS (pH 8.4), thus corresponding to the final sample.

Fluorescent liposomes were prepared using the same methodology. In this case, rhodamine covalently linked to phosphatidyl ethanolamine (Rho-PE) obtained from Avanti Polar Lipids, USA was included in the lipid composition at 0.1 mol% related to total lipid.

Liposomal formulations were characterized in terms of lipid and SQV contents and by the following incorporation parameters: initial and final SQV to lipid ratios ((SQV/Lip)i and (SQV/Lip)f, respectively. The Encapsulation Efficiency (E.E.) was defined as the percentage of:

[(SQV/Lip)f ]/[(SQV/Lip)i] × 100.

SQV incorporated in liposomes was determined by high-performance liquid chromatography (HPLC), as described in [56]. The HPLC system was an ELITE LaChrom Hitachi (Tokyo, Japan), comprising an L-2130 pump module, a Diode-Array L-2455 detector, and an autosampler L-2200 with a loop of 100 μL. The wavelength of the detector was set at 240 nm. The system was connected to a computer with specific software, Ez Chrom Elite, for integration and treatment of chromatograms. The analytical column was a LiChroCART^®^ (250-4,6) Purospher^®^ Star RP-8 (5 μm) (Merck, KGaA, Darmstadt, Germany) equipped with the respective guard column. The mobile phase, in an isocratic solvent system, was a mixture of 10 mM ammonium acetate buffer–acetonitrile (ACN) (35:65, *v*/*v*) pumped at a flowrate of 1.0 mL/min at 30 °C.

A calibration curve of SQV standards solubilized in acetonitrile, ranging from 80 to 1200 ng/mL, was used. Liposomal SQV samples were diluted in ACN in order to be within the range of the calibration curve. 

The Lipid content was determined using an enzyme-linked colorimetric method, Phospholipids Choline Oxidase-Peroxidase (Spinreact, St. Esteve de Bas, Spain).

The mean sizes of the liposomes were determined by dynamic light scattering, using a Zetasizer Nano S, Malvern Instruments, Malvern, UK, at a standard laser wavelength of 663 nm. The system also reports a polydispersity index, as a measure of particle size distribution, ranging from 0.0 for an entirely monodisperse sample up to 1.0 for a polydisperse suspension. Zeta potential of liposomal formulations was measured in a hydrodynamic sizing system, using a Zetasizer Nano Z (Malvern Instruments, Malvern, UK).

### 4.2. Cell Isolation and Culture Conditions

Human monocyte-derived macrophages (HMDM) were obtained from buffy-coats of healthy human donors provided by the national blood institute (Instituto Português do Sangue e da Transplantação, I.P., Lisbon, Portugal) following a previously described protocol [16].

### 4.3. Bacterial Cultures

*M. tuberculosis* H37Rv (American Type Culture Collection [ATCC] 27294), H37Rv GFP-expressing strain, and the clinical strains isolated from patients with active TB were grown in Middlebrook’s 7H9 medium supplemented with 10 % Oleic Acid Albumin Dextrose (OADC) enrichment (BD Difco, Franklin Lakes, NJ, USA), 0.02% glycerol, and 0.05% tyloxapol (Merck, KGaA, Darmstadt, Germany) at 37 °C. The clinical strains were provided and characterized by the TB National Reference Laboratory from the Portuguese National Institute of Health’s Dr. Ricardo Jorge (INSA). The clinical strain (INSA code 33427) is susceptible to streptomycin, isoniazid, rifampicin, and pyrazinamide (PZA); the MDR strain (INSA code 34192) is resistant to all those antibiotics plus ethionamide; the XDR strain (INSA code 163761) is resistant to all the previous antibiotics plus amikacin, kanamycin, capreomycin, moxifloxacin, and ofloxacin. All experimental procedures using live Mtb were performed in the Biosafety Level 3 laboratory at the Faculty of Pharmacy of the University of Lisbon, respecting the national and European containment level 3 laboratory management and biosecurity standards, based on applicable EU Directives. 

### 4.4. Macrophage Infection and Treatment

To obtain a single-cell bacterial inoculum, the bacterial cultures on exponential growth phase were centrifuged and washed in phosphate-buffered saline (PBS) and resuspended in macrophage culture medium without antibiotics. Clumps of bacteria in suspension were dismantled by ultrasonic bath for 5 min. Residual clumps were removed by low-speed centrifugation at 500× *g* for 1 min. Single-cell suspension was verified by fluorescence microscopy and quantified by optical density at 600 nm. The infection was performed with a multiplicity of infection (MOI) of one bacterium per macrophage. After 3 h of infection at 37 °C and 5% CO_2_, the cells were washed with PBS to remove free bacteria and cultivated in fresh complete medium. At this stage, the treatments with SQV-loaded liposomes or free SQV were applied at the selected concentrations of 50, 20, and 10 µg/mL. Unloaded liposomes or DMSO solvent were used as controls. For quantitative and qualitative analysis of liposome treatments by flow cytometry and fluorescence microscopy, rhodamine-labelled liposomes were used. After 3 h, the medium was renewed without treatments, except for the viability and cell death experiments, where the treatments were prolonged until the time-point of analysis.

### 4.5. Flow Cytometry

For quantification of intracellular rhodamine-labelled liposomes, macrophages were detached using Accutase and fixed in 4% paraformaldehyde for 1 h before being analyzed in a Cytek Aurora flow cytometer (Cytek Biosciences, Fremont, CA, USA). For cell death analysis, cells were stained with Apotracker Green and Zombie Red dyes (Biolegend, San Diego, CA, USA) prior to fixation, according to the manufacturer’s instructions. Data analysis was performed in FCS Express 7 (De Novo Software, Pasadena, CA, USA).

### 4.6. Fluorescence Microscopy

Fluorescence microscopy was performed in live cells, counter-stained with Hoechst 33342 dye (Thermo Fisher Scientific, Waltham, MA, USA), directly on the culture plates. Images were captured using a Zeiss Axio Observer microscope and analyzed in Zen 3.6 Blue Edition software (Carl Zeiss Microscopy, Jena, Germany).

### 4.7. Macrophage Viability

Cultures were incubated with 10% (*v*/*v*) PrestoBlue (Invitrogen, Carlsbad, CA, USA) resazurin-based solution at 37 °C and 5% CO2, according to the manufacturer’s instructions. After 3 h of incubation, fluorescence emission was analyzed in a Tecan M200 spectrofluorometer. Non-treated macrophages were used as reference for 100% viability, and puromycin-treated macrophages (5 µg/mL) were used as reference for 0% viability.

### 4.8. Proteolytic Activity

Following 24 h of infection, macrophages in a 96-well plate were washed with PBS and incubated in PBS with 30 µg/mL omnicathepsin (Z-FR-AMC, Z-Phe-Arg-AMC) (Enzo Life Sciences, Exeter, UK) fluorogenic substrate for 1.5 h at 37 °C in a Tecan M200 spectrofluorometer (Tecan Group, Männedorf, Switzerland). Fluorescence readings were performed every 5 min. Assay specificity was verified by incubating the cells with omnicathepsin substrate plus the general protease inhibitor E-64d (Merck, KGaA, Darmstadt, Germany) at 5 µg/mL.

### 4.9. Bacteria Intracellular Survival

Infected macrophages were lysed with 0.05% Igepal solution (Merck, KGaA, Darmstadt, Germany) for 15 min after 3 h of infection (T = 0) and after 1, 4, and 7 days of infection. Serial dilutions of the resulting bacterial suspension were plated in Middlebrook 7H10, supplemented with 10% OADC (BD Difco, Franklin Lakes, NJ, USA), and incubated for 2–3 weeks at 37 °C until colonies could be observed and counted under the microscope.

### 4.10. Statistical Analysis

Statistical analysis was conducted in GraphPad Prism 9 (GraphPad Software, San Diego, CA, USA). Multiple group comparisons were performed using one-way ANOVA followed by a Holm–Sidak post-hoc test. Differences were considered significant when the calculated adjusted-*p* value was equal to or below the alpha level of 0.05.

## Figures and Tables

**Table 1:** Physicochemical characterization of liposomes unloaded or incorporating saquinavir.

Formulation	Lipid Composition (Molar Ratio)	(SQV /Lip)i (μg/μmol)	(SQV /Lip)f (μg/μmol)	E.E. (%)	Ø (nm) (P.I.)	ζ Pot (mV)
Loaded liposomes	DOPC: DOPG (8:2)	60.9 ± 2.7	53.4 ± 4.9	90.3 ± 6.7	116 ± 1 (<0.1)	−23 ± 2
Unloaded liposomes	DOPC: DOPG (8:2)	na	na	na	113 ± 1 (<0.1)	−23 ± 1
Liposomes labelled with rhodamine Rho-PE (0.1 mol%)						
Loaded liposomes	DOPC: DOPG (8:2)	42.7 ± 0.4	47.7 ± 0.7	109.1 ± 0.8	114 ± 1 (<0.1)	−24 ± 2
Unloaded liposomes	DOPC: DOPG (8:2)	na	na	na	111 ± 1 (<0.1)	−25 ± 2

DOPC—dioleoyl phosphatidyl choline; DOPG—dioleoyl phosphatidyl glycerol; SQV—saquinavir; lip—lipid; (SQV/Lip)i—initial saquinavir to lipid ratio; (SQV/Lip)f—final saquinavir to lipid ratio; E.E. (%) = (SQV/Lip)f /(SQV/Lip)i × 100; Ø—mean size; P.I.—polydispersity index; ζ Pot—zeta potential; na—not applicable.

## Data Availability

Not applicable.
